# Causal evidence in health decision making: methodological approaches of causal inference and health decision science

**DOI:** 10.3205/000314

**Published:** 2022-12-21

**Authors:** Felicitas Kühne, Michael Schomaker, Igor Stojkov, Beate Jahn, Annette Conrads-Frank, Silke Siebert, Gaby Sroczynski, Sibylle Puntscher, Daniela Schmid, Petra Schnell-Inderst, Uwe Siebert

**Affiliations:** 1 Institute of Public Health, Medical Decision Making and Health Technology Assessment, Department of Public Health, Health Services Research and Health Technology Assessment, UMIT TIROL – University for Health Sciences, Medical Informatics and Technology, Hall i.T., Austria; 2 Centre for Infectious Disease Epidemiology & Research, University of Cape Town, South Africa; 3 Division of Health Technology Assessment, ONCOTYROL – Center for Personalized Cancer Medicine, Innsbruck, Austria; 4 Center for Health Decision Science, Departments of Epidemiology and Health Policy & Management, Harvard T.H. Chan School of Public Health, Boston, MA, USA; 5 Program on Cardiovascular Research, Institute for Technology Assessment and Department of Radiology, Massachusetts General Hospital, Harvard Medical School, Boston, MA, USA

**Keywords:** causal inference, health decision science, epidemiology, decision-analytic modeling, medical decision making, health technology assessment

## Abstract

**Objectives:**

Public health decision making is a complex process based on thorough and comprehensive health technology assessments involving the comparison of different strategies, values and tradeoffs under uncertainty. This process must be based on best available evidence and plausible assumptions. Causal inference and health decision science are two methodological approaches providing information to help guide decision making in health care. Both approaches are quantitative methods that use statistical and modeling techniques and simplifying assumptions to mimic the complexity of the real world. We intend to review and lay out both disciplines with their aims, strengths and limitations based on a combination of textbook knowledge and expert experience.

**Methods:**

To help understanding and differentiating the methodological approaches of causal inference and health decision science, we reviewed both methods with the focus on aims, research questions, methods, assumptions, limitations and challenges, and software. For each methodological approach, we established a group of four experts from our own working group to carefully review and summarize each method, followed by structured discussion rounds and written reviews, in which the experts from all disciplines including HTA and medicine were involved. The entire expert group discussed objectives, strengths and limitations of both methodological areas, and potential synergies. Finally, we derived recommendations for further research and provide a brief outlook on future trends.

**Results:**

Causal inference methods aim for drawing causal conclusions from empirical data on the relationship of pre-specified interventions on a specific target outcome and apply a counterfactual framework and statistical techniques to derive causal effects of exposures or interventions from these data. Causal inference is based on a causal diagram, more specifically, a directed acyclic graph (DAG), which encodes the assumptions regarding the causal relations between variables. Depending on the type of confounding and selection bias, traditional statistical methods or more complex g-methods are needed to derive valid causal effects. Besides the correct specification of the DAG and the statistical model, assumptions such as consistency, positivity, and exchangeability must be checked when aiming at causal inference. Health decision science aims for guiding policy decision making regarding health interventions considering and balancing multiple competing objectives of a decision based on data from multiple sources and studies, for example prevalence studies, clinical trials and long-term observational routine effectiveness studies, and studies on preferences and costs. It involves decision analysis, a systematic, explicit and quantitative framework to guide decisions under uncertainty. Decision analyses are based on decision-analytic models to mimic the course of disease as well as aspects and consequences of the intervention in order to quantitatively optimize the decision. Depending on the type of decision problem, decision trees, state-transition models, discrete event simulation models, dynamic transmission models, or other model types are applied. Models must be validated against observed data, and comprehensive sensitivity analyses must be performed to assess uncertainty. Besides the appropriate choice of the model type and the valid specification of the model structure, it must be checked if input parameters of effects can be interpreted as causal parameters in the model. Otherwise results will be biased.

**Conclusions:**

Both causal inference and health decision science aim for providing best causal evidence for informed health decision making. The strengths and limitations of both methods differ and a good understanding of both methods is essential for correct application but also for correct interpretation of findings from the described methods. Importantly, decision-analytic modeling should be combined with causal inference when developing guidance and recommendations regarding decisions on health care interventions.

## Zusammenfassung

**Zielsetzungen:**

Die Entscheidungsfindung im Gesundheitswesen ist ein komplexer Prozess, der auf gründlichen und umfassenden Bewertungen von Gesundheitstechnologien beruht und den Vergleich verschiedener Strategien, Werte und Kompromisse unter Unsicherheit beinhaltet. Dieser Prozess muss auf den besten verfügbaren Erkenntnissen und plausiblen Annahmen beruhen. Kausalinferenz und Entscheidungsanalyse sind zwei methodische evidenzbasierte Ansätze, die Informationen liefern, um die Entscheidungsfindung im Gesundheitswesen zu unterstützen. Bei beiden Ansätzen handelt es sich um quantitative Methoden, die statistische Techniken und Modellierungstechniken sowie vereinfachende Annahmen verwenden, um die Komplexität der realen Welt zu imitieren. Wir beabsichtigen in dieser Publikation, auf der Grundlage von Lehrbuchwissen und Expertenerfahrung beide Disziplinen mit ihren Zielen, Stärken und Grenzen darzustellen.

**Methoden:**

Um das Verständnis und die Unterscheidung der methodischen Ansätze der kausalen Inferenz und der gesundheitswissenschaftlichen Entscheidungsanalyse zu erleichtern, haben wir beide Methoden mit dem Schwerpunkt auf Ziele, Forschungsfragen, Methoden, Annahmen, Grenzen und Herausforderungen sowie verfügbare Software untersucht. Für jeden methodischen Ansatz setzten wir eine Gruppe von vier Experten aus unserer eigenen Arbeitsgruppe ein, um jede Methode sorgfältig zu begutachten und ihre Charakteristika zusammenzufassen. In den darauffolgenden strukturierten Diskussionsrunden und schriftlichen Überprüfungen waren Experten aus allen Disziplinen einschließlich Health Technology Assessment (HTA) und Medizin beteiligt. Die gesamte Expertengruppe diskutierte Ziele, Stärken und Grenzen der beiden methodischen Bereiche und mögliche Synergien. Abschließend wurden Empfehlungen für die weitere Forschung abgeleitet und ein kurzer Ausblick auf zukünftige Trends gegeben.

**Ergebnisse:**

Methoden der kausalen Inferenz zielen darauf ab, kausale Schlussfolgerungen aus empirischen Daten über die Beziehung zwischen vorher festgelegten Interventionen und einem bestimmten Zielendpunkt zu ziehen. Es werden ein kontrafaktischer Rahmenansatz und statistische Techniken angewandt, um kausale Auswirkungen von Expositionen oder Interventionen aus diesen Daten abzuleiten. Die kausale Inferenz basiert auf einem Kausaldiagramm, genauer gesagt auf einem *directed acyclic graph *(DAG), der die Annahmen bezüglich der kausalen Beziehungen zwischen Variablen darstellt. Je nach Art des Confounding und des Selektionsbias sind traditionelle statistische Methoden oder komplexere g-Methoden erforderlich, um gültige kausale Effekte abzuleiten. Neben der korrekten Spezifikation des DAG und des statistischen Modells müssen Annahmen wie *Consistency*, *Positivity* und *Exchangeability* überprüft werden, wenn man kausale Schlüsse ziehen möchte. Die gesundheitswissenschaftliche Entscheidungsanalyse zielt darauf ab, die politische Entscheidungsfindung in Bezug auf Gesundheitsmaßnahmen zu unterstützen, wobei mehrere konkurrierende Ziele einer Entscheidung auf der Grundlage von Daten aus verschiedenen Quellen und Studien berücksichtigt und abgewogen werden. Diese Studien umfassen z. B. Prävalenzstudien, klinische Studien und langfristige Routinebeobachtungsstudien zur Wirksamkeit sowie Studien zu Präferenzen und Kosten. Die Entscheidungsanalyse bietet einen systematischen, expliziten und quantitativen Rahmen, um Entscheidungen unter Unsicherheit zu strukturieren. Entscheidungsanalysen basieren auf entscheidungsanalytischen Modellen, die den Krankheitsverlauf sowie die Aspekte und Folgen der Intervention nachbilden. Je nach Art des Entscheidungsproblems werden Entscheidungsbäume, Zustands-Übergangs-Modelle (z. B. Markov-Modelle), diskrete Ereignissimulationsmodelle, dynamische Übertragungsmodelle oder andere Modelltypen verwendet. Die Modelle müssen anhand von Beobachtungsdaten validiert werden, und es sind umfassende Sensitivitätsanalysen durchzuführen, um die Unsicherheit zu bewerten. Neben der angemessenen Wahl des Modelltyps und der validen Spezifikation der Modellstruktur muss geprüft werden, ob die Einflussparameter für die daraus resultierenden Auswirkungen als kausale Parameter im Modell interpretiert werden können. Andernfalls werden die Ergebnisse verzerrt.

**Schlussfolgerungen:**

Sowohl die kausale Inferenz als auch die gesundheitswissenschaftliche Entscheidungsanalyse zielen darauf ab, die beste kausale Evidenz für eine informierte Entscheidungsfindung zu liefern. Die Stärken und Grenzen beider Methoden sind unterschiedlich, und ein gutes Verständnis beider Methoden ist für die korrekte Anwendung, aber auch für die korrekte Interpretation der Ergebnisse der beschriebenen Methoden unerlässlich. Wichtig ist, dass entscheidungsanalytische Modellierungen mit kausalen Inferenzmethoden kombiniert werden, wenn es um die Entwicklung von Leitlinien und Empfehlungen für Entscheidungen über Interventionen im Gesundheitswesen geht.

## 1 Introduction

According to the World Health Organization (WHO), public health is “the science and art of promoting health, preventing disease, and prolonging life through the organized efforts of society” [1]. This involves many different disciplines, all of them aiming to protect the health of populations. Politicians and public health decision makers need to decide which health care programs are implemented based on thorough and comprehensive health technology assessments (HTA) [2], which evaluate the balance of benefits, harms, cost-effectiveness, ethical, legal, social and patient aspects, and given limited resources and diverse needs [3], [4], [5]. Such decisions are often complex and must usually be made under uncertainty, sometimes with imperfect knowledge and evidence, and in some cases under extreme time constraints [6]. Therefore, these decisions must rely on the best available evidence at the time of decision and they must apply the most rigorous methods. Among others, statisticians, epidemiologists and health decision scientists are involved in analyzing and summarizing the data in order to derive the potential causal consequences of alternative health technologies, that is, any actions representing possible choices under decision [3], [7].

Epidemiologists and statisticians provide information on disease occurrence and spread of the disease. Further, they investigate potential risk factors and effectiveness of interventions. This knowledge is usually gained from empirical studies. One area of epidemiology, causal inference, aims to draw conclusions on the causal effect of interventions (actions) from empirical data and prior knowledge. Identifying causal relations between the interventions (or “actions”) under investigation and the target outcomes of interest provides potential for actions to maintain or improve health [8]. Only if the relation between an action and an outcome is causal, the action under investigation will show the intended effect. In general, studies that are considered to have the lowest risk for bias are well-designed randomized clinical trials (RCT) [9]. However, the external validity and generalizability may be limited, since RCTs usually have strict inclusion criteria. Therefore, additional evidence from real-world observational studies is needed. Observational studies may suffer less from external validity but are much more prone to biases such as confounding and selection bias reducing internal validity. Such biases must be controlled for using the appropriate causal methods [10], [11], [12].

Health decision scientists usually do not only derive conclusions from primary studies but often (if not in most cases) synthesize information from different sources [4]. They typically lay out and analyze all aspects of a complex decision and identify the “optimal” choice of intervention. To achieve this goal, information on all aspects relevant to the decision problem is needed over a sufficiently long time horizon that includes all important consequences of such a decision. Important aspects include but are not limited to patient-relevant benefits in terms of morbidity, duration of disease, health-related quality of life and mortality as well as harmful unintended effects, cost-effectiveness and other aspects [13]. Such information is rarely available from one empirical database. Hence, health decision science identifies the best available data sources and combines these data in a decision-analytic model in order to simulate the causal effects of the compared interventions [5], [14], and transfers the existing evidence to the population of interest [14]. As models are simplifying the complex world, assumptions are required. A decision-analytic [14] study following best practice principles transparently lays out the simplifying assumptions which are part of the model structure [15], [16], [17], [18] as well as the input parameters for the model [19], [20]. Knowing that decisions must be made on the basis of the best available data and under uncertainty, the first choice of a health decision analyst is not to omit uncertainty, but rather identify the potential consequences of uncertainty [19], [20].

Both disciplines, causal inference epidemiology and decision-analytic modeling in health decision science, rely on assumptions and often very complex models. In order to see the strengths and limitations of both methodological approaches and to be able to judge the applicability and validity of each approach in the light of a specific decision problem, the key principles of both methods and related techniques must be known and their interrelation must be understood. However, most of the published literature either focuses on epidemiology or on health decision science, and therefore, even systematic reviews are not helpful when assessing the differences and potential synergies of both methods. Thus, in this scoping document, we intend to review and lay out both disciplines with their aims, strengths and limitations based on a combination of textbook knowledge and expert experience.

## 2 Methods

For this scoping document, we reviewed and summarized the methods of causal inference and health decision science. In order to address both differences and overarching concepts of these methods, we created a group of experts from our own working group. We chose two “bridging” experts with extensive expertise in both areas (FK, US) and we added three further experts with particular expertise in both causal inference (MS, IS, DS) and health decision science (BJ, ACF, GS) as well as a health technology assessment (HTA) expert (PSI) and an expert in medicine (SS). The expert groups in causal inference and in health decision science carefully reviewed and summarized the description of the respective method with a focus on the following predefined topics: aims, research questions, methods, assumptions, limitations and challenges, and software. Rather than performing a systematic literature search, the experts used their experience and expertise as well as their knowledge regarding common textbooks [5], [8], [10], [11], [21], [22], [23], [24], [25], [26], [27], [28], [29], methodological guidelines and key references [2], [4], [6], [12], [15], [16], [17], [18], [19], [20], [30], [31], [32], [33], [34], [35], [36], [37], [38], [39], [40], [41], [42], [43], [44], [45], [46], [47], [48], [49], [50], [51], [52], [53], [54], [55], [56], [57], [58], [59], [60], [61], [62], [63], [64], [65], [66], [67], [68] in the field to summarize and explain each method.

The next step involved structured discussion rounds and written reviews, in which the experts from all disciplines including HTA and medicine were involved. Both the causal inference and health decision science group presented the respective method. Questions were structured regarding content and scientific terminology and language. In the expert discussions, the questions were carefully answered and reasons for misunderstanding were debated. The improved explanation of the method was transformed into text and a common language for reporting, presenting and explaining each method was sought. The written word was again reviewed by the group of the respective other areas to make sure that the description is understood outside of the own scientific community.

In order to relate the theoretical methods to applied real world decision problems, we selected case examples from the medical literature. The chosen case examples were selected in a way that they served both the formal and intuitive understanding of causal effects of interventions aiming at typical exposures or medical treatments. We sought to use examples that required both causal inference and decision-analytic modeling to comprehensively answer the decision question.

Finally, the entire expert group discussed strengths and limitations of both methodological areas, and how these methods can be used in synergy with a focus on selected key issues of the case examples from the literature. Finally, we derived recommendations for further research and provide a brief outlook on future trends.

## 3 Results

### 3.1 Causal inference

#### 3.1.1 Causal aims and research questions

The goals of many research questions are causal in nature: will a new drug lead to lower 5-year mortality compared to the currently used drug? Does the use of a certain medical device improve quality of life (QoL) compared to not using the device? Would the implementation of a new government antismoking campaign in school decrease the rate of smoking? Such causal questions are always tied to an action, applied to a unit (such as a person): for example, a person can decide to apply an icepack on a sports injury and depending on whether the icepack is being used or not, we may observe a different amount of swelling the next day. If the respective person did actually use the ice, we may ask what would have happened if the person (contrary to the fact) had not used it. This is a hypothetical scenario which is counterfactual, that is, different from the “fact” that has actually been observed. The core of causal inference is to understand that causal questions relate to outcomes that are *counterfactual*, are therefore not observed, and – most importantly – cannot be calculated from the observed data distribution alone, exactly because a post-intervention distribution (that results from a change in action) is the one of interest.

The formalisms and notations used in causal inference often refer to counterfactuals [8]: let *A* be an intervention of interest (e.g., a drug) and *Y* be the outcome of interest (e.g., mortality), then Y_i_^a^ is the outcome for unit *i* that would have been observed if the unit had been exposed to action *a* (possibly contrary to the fact). Causal inference is typically not possible on an individual level; thus, estimands such as the *average treatment effect* (ATE),

E(Y^1^)–E(Y^0^), with E() being the expectation, and the superscripts denote the counterfactuals,

are of interest. The ATE compares the expected outcome that would have been observed if *every* unit had received the intervention *a=1*, compared to if every unit had received *a=0*. Similar estimands for binary interventions and binary outcomes are the causal risk ratio (RR) or causal odds ratio (OR) respectively:

RR=P(Y^1^=1)/P(Y^0^=1), where P() is the probability

OR=(P(Y^1^=1)/P(Y^1^=0))/(P(Y^0^=1)/P(Y^0^=0))

Above we asked whether applying an icepack on a sports injury reduces swelling. If *A* is binary and refers to the icepack (1, if used; 0 otherwise) and Y is the measured circumference of the knee with ordinary tape measure 24 hours after applying the icepack, then E(Y^1^)–E(Y^0^), that is, the ATE, is the estimand that corresponds to the scientific question asked.

It may sound trivial, but the first task in causal inference is to commit to a causal estimand that captures the scientific question of interest. Common estimands are ATE, RR and OR for binary interventions. For continuous interventions, so-called marginal structural models (MSM), which relate a *counterfactual* outcome with the intervention, are important, for example, E(Y^a^)=f(A), where f() is an arbitrary function. All these estimands may be *conditional* on a subset of the population, say smokers and non-smokers. Often, questions of effect modification are captured in such conditional estimands.

In summary, causal questions are inherently tied to actions/interventions that result in outcomes that are not always observed. Counterfactual notation is a language that can be used to translate a scientific question into a formal quantity. To precisely define such quantities, several decisions have to be made: definition of the target population, choice of variables to be intervened upon, type of intervention (one or many time points), outcome of interest and the choice of effect measure (e.g., risk ratio, MSM, possibly conditional on subgroups).

If one has committed to a *specific* scientific question, represented by a counterfactual estimand, causal inference requires:

1. A causal model, that is, a model which summarizes the *knowledge* on how the data has been generated.
2. An evaluation of whether in a given context the causal question can be answered; and if yes, what data and assumptions are required.
3. An appropriate statistical method.

In the next section, causal models are introduced.

#### 3.1.2 Directed acyclic graphs

**Figure 1:**
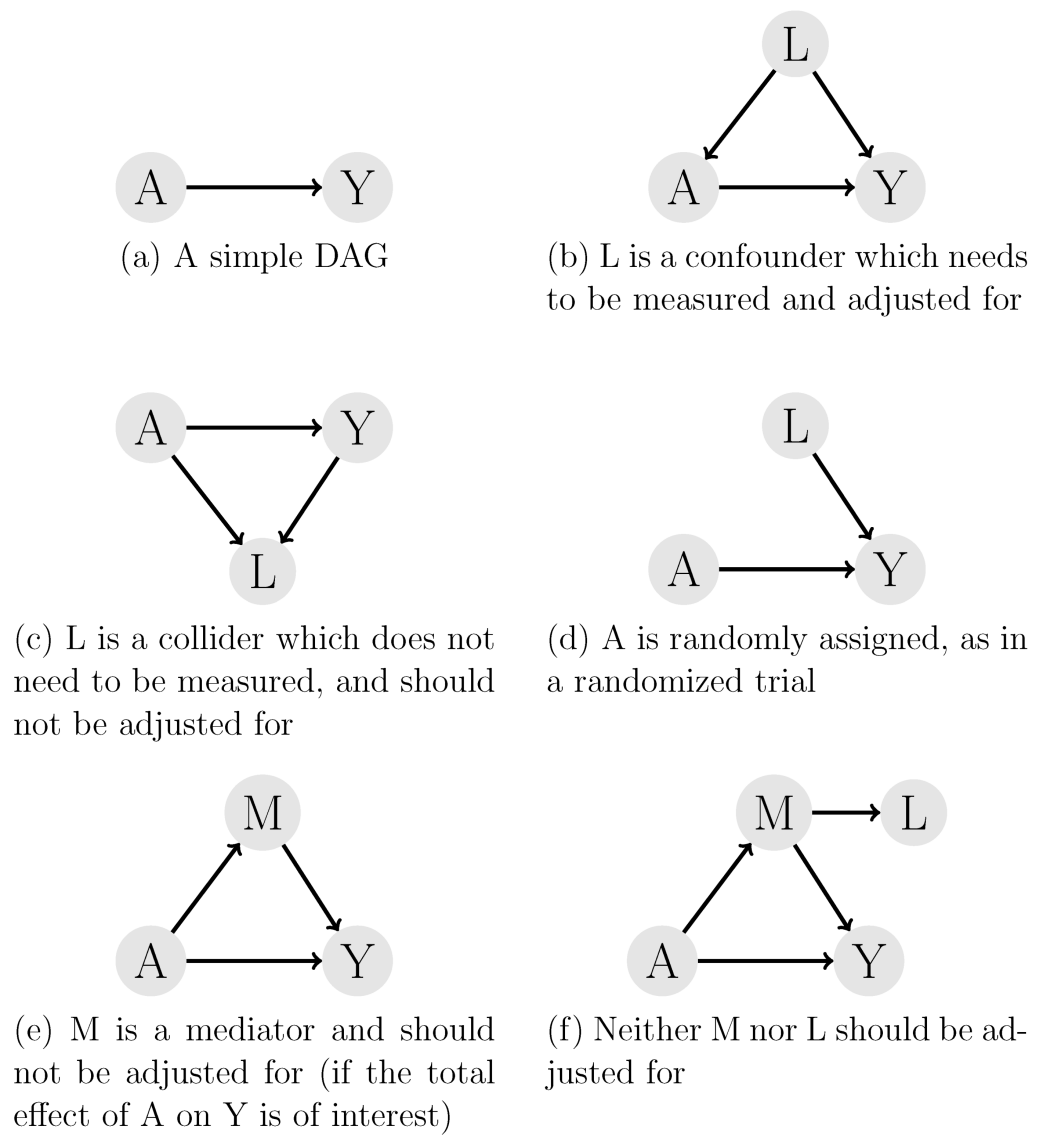
Examples of directed acyclic graphs (created with and following the layout of the software “daggity” [158])

Causal subject-matter knowledge can be expressed with directed acyclic graphs (DAGs), among other options (such as non-parametric structural equation models). In a DAG, each circle represents a variable (in this text, A represents an action and Y an outcome). An arrow from A to Y represents the knowledge or assumption that A causes Y (Figure 1a (Fig. 1)). More importantly, the absence of an arrow means we assume no causal relationship between the two respective variables. It is important to understand that DAGs are used to summarize and visualize the data-generating process of a natural causal process that exists independent of which data have been measured. As a consequence, variables in a DAG may be measured or unmeasured in particular studies.

In DAG language, concepts such as confounders, colliders and mediators are important. A *confounder* is a variable that causes two other variables, such as illustrated by the variable L in Figure 1b (Fig. 1). Note that L proceeds A and Y in terms of time, as otherwise it could not be a cause of both A and Y. A *collider* is a variable that is being caused by two other variables, see Figure 1c (Fig. 1). Here, A and Y precede L. A *mediator* or intermediate step M lies on the path between A and Y (see Figure 1e (Fig. 1)). These concepts are important to establish whether a particular causal question can be answered, and if yes, how, as outlined in the next section.

#### 3.1.3 Identification and assumptions: can the research question of interest be answered?

*Identification* means establishing whether for a given estimand (e.g., the ATE) and a given causal model encoded in a DAG, the causal question can be answered or not, and if yes, under what assumptions, and which variable should be used for controlling for confounding. The answer to this question lies in Pearl’s *back-door criterion* [23]: informally speaking, this criterion says that we need to (i) “block all back-door paths” from A to Y, and (ii) not control for “descendants” of *A* in the analysis. Let us clarify what is meant by this criterion. A *back-door path* is defined as path from A to Y that starts with an arrow into A (i.e., starting with A ← … as opposed to A → …). Consider Figure 1b (Fig. 1): here, A ← L → Y is a back-door path, where *L confounds* the effect of A on Y. The confounding generated by *L* can be removed by blocking that path, that is, by including L in the analysis (i.e., “adjusting for it”, see Section 3.1.4 below). A path is also blocked if it contains a collider (which is *not* “adjusted for”); for example the path A ← L→ L2 ← Y is a back-door path because it starts with an arrow into A and it is blocked because it contains a collider (*L2*). The path would be opened if *L2* was included in the analysis, but closed if both *L* and *L2* were included. Colliders can appear in many circumstances, and may even relate to missing data or censoring indicators. The interested reader is referred to well-known examples such as the obesity paradox [69], the smoking-preeclampsia paradox [70], the birthweight example [71], the sodium intake paradox [58], and survival bias [8], [72]. A descendant is a variable that results from *A*, that is, a variable *M* on paths such as A → M or A → … → M. A mediator is a descendant of A and conditioning (“adjusting for”) on mediators, or any of the mediator’s descendants, would be incorrect. Figure 1e (Fig. 1) and Figure 1f (Fig. 1) give examples.

In summary, Pearl’s back-door criterion typically tells us which variables to include in the analysis and which ones not. If we are interested in the effect of A on Y, measuring and conditioning on mediators (or descendants of the mediators) is incorrect. However, to close all back-door paths from A to Y, confounders are typically conditioned upon (adjusted for), if measured, while colliders are not supposed to be included in the analysis. As a consequence, unmeasured confounders may prohibit appropriate causal effect estimation, whereas unmeasured mediators or colliders may not necessarily be a problem.

Before estimating the quantity that relates to the scientific question of interest, it makes sense to reflect upon the assumptions that are usually quoted in the literature being necessary to conduct causal inference. In simple (single time point) settings, they can be expressed as follows:

1. Consistency: that is, if A_i_=a, then Y_i_^a^=Y_i_ , with i being the individual subjects
2. Positivity: that is, P(A=a|**L**=**l**) > 0 for all P(**L**=**l**)≠0
3. (Conditional) Exchangeability: Y^a^ independent of A|**L** for all A=a and **L**=**l**

Hernan and Robins [8] and Schomaker et al. [72] give the corresponding definitions for longitudinal setups. What do these assumptions mean? Informally, conditional exchangeability refers to comparability of the compared arms and is met if all back-door paths can be blocked by adding the respective confounders as “adjustment variables” in the analysis (see below) [44]. Thus, inspection of the DAG and evaluation of what variables have been measured leads to a statement on whether conditional exchangeability is likely met or not. Note that this assumption can*not* be tested from the data. Consistency is a technical requirement to link the observed data to the counterfactual. It may however be violated if an intervention is not well-defined or multiple versions of the intervention exist. For example, if a surgery can be performed in multiple ways, then the link between the surgery (A=1) and the counterfactual outcome (Y^a=1^, or briefly Y^1^) is not clear, as different versions of the interventions may lead to different counterfactual outcomes. There are many subtleties around this assumption that is often viewed as a theorem rather than an assumption; see the literature for a thorough discussion [51], [73], [74], [75]. Positivity requires a positive probability of treatment assignment across all covariate strata. In a finite data set, with a couple of (possibly continuous) covariates, there will often be some violations. However, the positivity assumption can sometimes be relaxed, especially if appropriate “smoothing” methods are used (see below).

Note that randomized experiments typically fulfil the above assumptions by design: positivity is guaranteed as by definition P(A=a)>0; consistency is not an issue if the study protocol is unambiguous about the intervention; and exchangeability is guaranteed as well. Therefore, randomized experiments do not face the problem of confounding (neither measured nor unmeasured), see also Figure 1d (Fig. 1). However, if randomized experiments face practical issues such as non-adherence to treatment assignment, or treatment switching, measurement error or drop out, additional corrections may be required or causal inference may be impossible. The interested reader is referred to the literature [8], [46], [48], [49].

Below, we are now going to introduce statistical methods that are suitable for causal inference from observational data (or imperfect randomized experiments).

#### 3.1.4 Estimation: the statistical model

**Figure 2:**
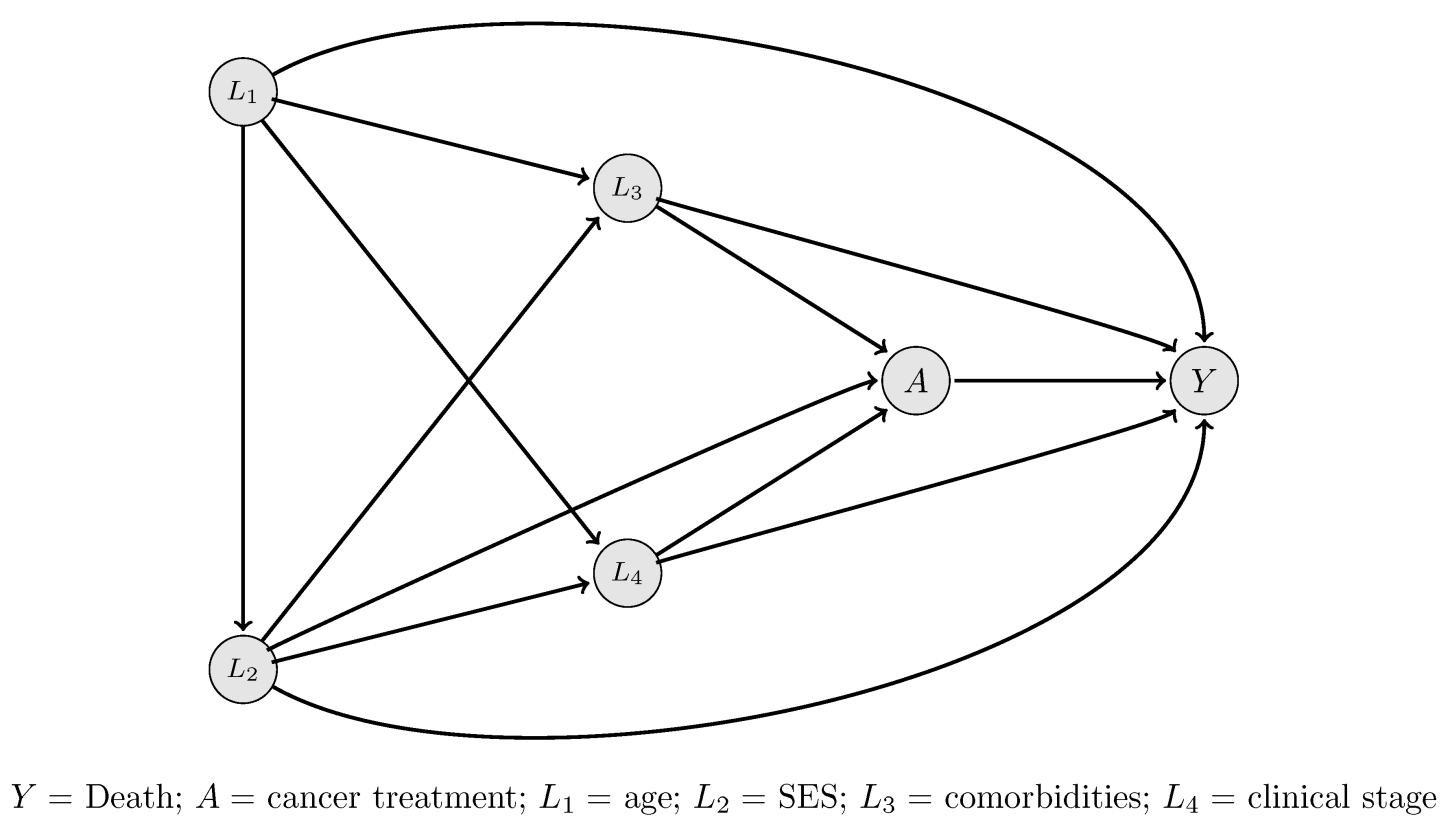
Directed acyclic graph for cancer

To illustrate appropriate statistical methods, we are introducing an example. We are looking at an example from cancer epidemiology; see Luque-Fernandez et al. [59], [76]. In this example, we are interested in the effect of dual treatment therapy (radio- and chemotherapy), compared to single therapy (chemotherapy only) on the probability of one-year survival among colorectal cancer patients, that is, the estimand of interest is P(Y^1^=1)/P(Y^0^=1). We know that there are confounders which affect both treatment assignment and the outcome, namely clinical stage, socioeconomic status, comorbidities, and age. Evidence shows that older patients with comorbidities have a lower probability of being offered more aggressive treatments and therefore they usually get less effective curative options. Also, colorectal cancer patients with lower socioeconomic status have a higher probability of presenting with an advanced clinical stage at initial diagnosis, thus they usually get offered only palliative treatments. This knowledge is represented in the DAG shown in Figure 2 (Fig. 2).

The causal DAG in Figure 2 (Fig. 2) tells us that there are no mediators or colliders that would need to be taken into account when estimating the effect of cancer treatment on mortality. There are, however, various back-door paths that start with arrows into the treatment variable. They can be blocked if the variables of **L**=(age, socioeconomic status [SES], comorbidities, stage) are included and adjusted for in the analysis.

We now introduce four causal inference methods to estimate this effect if the data on all variables has been measured [8], [11]. Other methods are briefly commented on at the end of this paragraph.

##### 3.1.4.1 The g-formula

This method integrates out the confounders, with respect to the post-intervention distribution. If **L** is discrete we can state it as:

E(Y^a^)=∑_l_ E(Y|A=a,L=l) x P(L=l)

This equality holds under the abovementioned assumptions of conditional exchangeability, positivity and consistency. In our example, where E(Y^a^)=P(Y^1^=1), we can proceed as follows:

- *Step 1*. Estimate a logistic regression model for the conditional expectation E(Y|A=a,**L**=**l**), that is, P(Y=1|A,**L**).
- *Step 2*. Following the time-order, create a new data set where **L** is estimated by the empirical distribution (i.e., filled in with the observed data) and A is intervened upon, that is, set as A=1 (for every unit).
- *Step 3*. Then, using the estimated regression model from step 1 and the new (post intervention) data from step 2, predict the outcome under this setup. Take the mean of the predicted outcome as an estimate for E(Y^1^)=P(Y^1^=1).
- *Step 4*. Repeat steps 2 and 3 for A=0, to obtain an estimate for E(Y^0^)=P(Y^0^=1).
- *Step 5*. Now, the causal risk ratio P(Y^1^=1)/P(Y^0^=1) or the causal risk difference P(Y^1^=1)–P(Y^0^=1) or any other effect measure can be estimated using the estimates from above.
- *Step 6*. Use bootstrapping to obtain confidence intervals.

In our example above, using the simulated data from Luque-Fernandez and a regression including main effects and interactions of treatment and SES and stage, we obtain a causal risk ratio of 0.46 (95% CI: 0.41; 0.52). This means the risk if everyone had received dual therapy is 0.46 times the risk of everyone receiving monotherapy (under the above mentioned assumptions).

The g-formula was first applied in a doctoral thesis of Siebert assessing risk factor intervention on coronary heart disease (CHD) under the supervision of Robins and co-supervision of Hernán in a collaboration project with the World Health Organization (WHO) [77], [78].

##### 3.1.4.2 Inverse probability of treatment weighting (IPTW)

This method uses weighting in order to achieve conditional exchangeability within the strata of the confounders. The weighted population is a pseudo population in which there is no confounding. Under the abovementioned assumptions of conditional exchangeability, positivity and consistency, and for a binary intervention, it holds that

E(Y^a^)=E(Y×I(A=a)/P(A=a|**L**=**l**).

IPTW can be implemented in many ways. For example, in the cancer example we can do the following:

- *Step 1*. Estimate the intervention assignment mechanism P(A=1|**L**=**l**) using logistic regression.
- *Step 2*. For those units that actually received the treatment (I(A=1)), predict the probability P(A=1|**L**=**l**) from the regression model of step 1.
- *Step 3*. To estimate E(Y^1^)=P(Y^1^=1), use a weighted mean of the observed outcomes, where the weights are the inverse predicted probabilities for those units where A=1, and 0 otherwise.
- *Step 4*. Repeat steps 2 and 3 to estimate E(Y^0^)=P(Y^0^=1).
- *Step 5*. Now, the causal risk ratio P(Y^1^=1)/P(Y^0^=1) can be estimated using the estimates from above.
- *Step 6*. Use bootstrapping or robust standard errors to obtain confidence intervals.

In our example above, we obtain a causal risk ratio of 0.47 (95% CI: 0.40; 0.55).

Marginal structural models with IPTW were first applied in (2000) by Hernán and colleagues assessing the causal effect of zidovudine on the survival of HIV-positive men [45].

##### 3.1.4.3 Nested structural models with g-estimation

This is a semiparametric method that estimates the potential outcome for each individual using a causal (i.e., structural) model and correlates these potential outcomes with the observed intervention/exposure variable within levels of confounders L, using the assumption of no unmeasured confounding (ANUC). The parameters of the causal model that yield a zero correlation between the potential outcomes and the observed intervention/exposure within levels of confounders L are the “true” model parameters.

- *Step 1*. Choose a causal model structure (e.g., additive, multiplicative) and keep the causal effect measure(s) as (a) parameter(s) in this model.
- *Step 2*. For each subject in the dataset, calculate the potential outcome Y^a=0^ “backwards” from the observed outcome Y by “removing” the intervention effect in those with the observed intervention A=1 for each set of causal model parameters within strata of L.
- *Step 3*. Find the counterfactual outcome so that the observed intervention A is independent of the potential outcome Y^a=0^ given confounders L (i.e., minimize correlation, maximize p value).
- *Step 4*. Use the effect estimate from the model identified in step 3 as the intervention’s causal effect estimate.

Nested structural models with g-estimation were first applied in (1992) by Robins assessing the causal effect of prophylaxis therapy for Pneumocystis carinii pneumonia on the survival of AIDS patients [62].

##### 3.1.4.4 Regression

Another popular option, which works under specific circumstances, is to use regression techniques, and interpret the regression coefficients causally. In the cancer example, a logistic regression model (which includes the intervention and all covariates) leads to an odds ratio of 0.31 (95% CI: 0.26; 0.37). A Poisson regression leads to an estimated risk ratio of 0.46 (95% CI: 0.40; 0.53).

While using regression coefficients for causal effect estimation is common, it comes with two caveats: first, causal effect estimation for many longitudinal setups is invalid (see below); and second, regression targets are by definition *conditional* effect estimands, while often marginal quantities are of interest, as in our example. In general, there is no guarantee that marginal and conditional estimates are identical, for example, when the effect measure (e.g. odds ratio) is not collapsible or under effect modification; see Luque-Fernandez et al. for a thorough discussion and illustration of this phenomenon [76].

Traditional regression analysis has first been applied by Legendre in 1805 [79] and by Gauss in 1809 [80] in the field of astronomy to derive the orbits of comets around the sun. Regression analysis has been used innumerable times in the evaluation of interventional effects in health sciences, economics and other fields [27].

G-formula, IPTW and g-estimation are summarized under the term “g-methods”. G-methods can be extended to the longitudinal setup, where interventions are time-varying and occur at multiple time points. Details on g-methods can be found in the comprehensive online textbook of Hernán and Robins [8] and further comprehensive material from the literature [31], [32], [42], [68], [72], [81]. An important point to highlight in the context of causal inference is that in the presence of time-varying confounding, the use of naïve regression may give invalid results and g-methods must be used [36]. Time-varying confounding occurs when confounders are simultaneously affected by the treatment or exposure of interest. That is, in our terminology: a confounder L_t_ affecting A_t_ is also affected by a prior intervention A_t–1_ such that the following causal chain is resulting: A_t–1_ → L_t_ → A_t_ → L_t+1_ → A_t+1_ → L_t+2_ → … etc. [8].

#### 3.1.5 Limitations and challenges

The use of causal inference comes with many challenges. Some of the key issues are described in the following. First, from a statistical perspective, model specification is crucial: for IPTW, the intervention assignment mechanism needs to be modeled correctly, possibly at each time point for longitudinal settings. For the application of g-estimation in settings where treatment differs over time (e.g., treatment is “on” during some times and “off” during others), the assumption of common effects must be made, which may or may not be valid [62]. For the g-formula, the outcome model as well as confounder models for the longitudinal setting need to be modeled correctly [8], [11], [61], [78]. It is likely that models are misspecified in many applications and hence effect estimates may be biased. To overcome this problem, doubly robust estimators, such as targeted maximum likelihood estimation (TMLE), have been developed. They allow for the integration of machine learning algorithms while retaining valid statistical inference. This is typically not the case for IPTW, regression, and the g-formula. The brief idea of TMLE is as follows: first, the data are standardized with respect to L, as with the g-formula. Then, the intervention assignment mechanism is used to update the g-formula estimate. This may then reduce bias, or narrow the confidence interval limits, if no bias is existent. Unbiased estimation is possible, even if one of the two models (for Y or A) is misspecified. Tutorials and software for TMLE methods are available [59], [82], [83], [84].

Second, coming up with a meaningful and well-justified DAG is a general challenge, independent of which causal statistical method is used. Some progress has been made with respect to this topic in the field of “causal discovery”, that is, searching and learning the DAG from data and additional assumptions (e.g., about time sequence) [85], [86]. However, it remains unclear to what degree DAGs derived with causal search algorithms from data will be a feasible and robust option for deriving DAGs from data [87].

Third, violations of the positivity assumption are common. Approaches such as IPTW are particularly sensitive to such violations, while the g-formula and TMLE in combination with machine learning are less prone to such problems [88]. Nevertheless, the development of more robust approaches is an active area of research [89].

#### 3.1.6 Software

All mentioned methods (IPTW, g-formula, g-estimation, regression, TMLE) can be implemented manually in standard statistical software (e.g., SAS, Stata, R). Guidance is given in the respective tutorials [42], [59]. A flexible software available is the *ltmle* package for the statistical software R, which can be used for deriving IPTW, g-formula and LTMLE estimates, for cross-sectional and longitudinal data. It integrates estimation with machine learning and allows survival analysis [83]. A similar package, *tmle*, offers the same features, but not for longitudinal data [82]. A recent R-package, *gfoRmula* [90], handles a variety of settings, including longitudinal data with competing risks. There is also a good Stata routine for implementing the g-formula [37]. In Stata, an implementation for TMLE is available, too [26], [28], [29], [91], [92].

### 3.2 Health decision science 

#### 3.2.1 Health decision science aims and research questions

The main aim in applications of health decision science is to guide clinical or public health decisions based on evidence and prior knowledge. Clinical and public health decisions are complex and involve many different aspects, values and trade-offs, and must usually be made under uncertainty.

According to the Encyclopedia of Medical Decision Making [93], one of the most important tasks of health decision analysts is to derive causal interpretations from decision-analytic models. In such models, an intervention, strategy, action, or risk factor profile is modeled to have a causal effect on one or more model parameters (e.g., probability, rate, or mean), which influence the outcome such as morbidity, mortality, quality of life, etc.

Decisions may have to be made on the level of individuals, subgroups or the entire population: What is the optimal personalized treatment strategy for a specific patient with specific characteristics? Should a screening program be offered to a specific population? Should a new drug be covered by the national health insurance? Should the government introduce a mandatory policy of face mask wearing in the light of an infectious disease outbreak?

On an individual level, aspects driving such a decision may be the individual’s well-being, the expected course of the disease, the expected quality of life, potential benefits, potential risks or side effects, own preferences, etc. On the societal level, different aspects may trigger the decision. Besides the benefits, any intervention may induce potential risks or harms, demand to the caregiver, costs and have ethical, legal and social implications. Decisions may include multiple strategies, and single time point interventions, complex treatment algorithms, or entire programs. These potential strategies must be well defined.

Health decision science uses a method known as decision analysis, which informs decisions on choices regarding multiple objectives [94]. Decision analysis uses decision-analytic models and simulation techniques to derive incremental benefit-harm or cost-effectiveness ratios when comparing different interventions or health technologies [3], [4], [5], [95]. Other terms with the same or similar meaning include “computer simulation”, “mathematical models”, or “agent-based models”. Decision analysis is a quantitative systematic approach that aims to (1) explicitly lay out all aspects of a decision, (2) balance all elements of the decision, (3) identify the “optimal” decision based on a-priori defined criteria and concepts (e.g., utilitarianism), and (4) provide a structured basis for discussion. Health decision science is not the art of automatically making the decision without human involvement [3], [4], [5], [95].

A formal decision analysis includes (1) a well-defined research question, (2) a decision-analytic model, (3) valid model input parameters, outcomes, and preferences, (4) an analytic time horizon, (5) validation, (6) base-case analysis, and (7) evaluation of uncertainty (sensitivity analysis) [15], [16], [17], [18].

The first step of a decision analysis is to structure the decision problem itself. This includes identifying the research question. In trials, we are used to the PICO framework, where P stands for population, I for intervention, C for comparator, and O for outcome. Besides all those aspects, the research question in health decision science needs to include the perspective and the time horizon. Therefore, in analogy to prognostic studies, one could request a PICOST framework, which includes also time horizon and setting. The optimal strategy may differ depending on the outcome chosen, the perspective adopted, and the willingness to pay elicited (see section 3.2.6), and the time frame considered.

The simulated population should reflect the target population for which the decision is intended to be made, that is, patient characteristics and the respective healthcare setting, country, etc.

We need to lay out all relevant intervention choices regarding interventional strategies, including the current standard of care, to obtain a list of suitable “comparators”. These interventions may involve policies, complex treatment strategies with dynamic testing and treatment algorithms, drug treatments, nonpharmaceutical interventions (such as quarantine), surgeries or complex multidisciplinary programs. When contrasting the alternative choices, we follow the counterfactual approach, that is, we compare a world where choice A is made to a world where choice B is made, to a world where choice C is made, etc.

In health decision science, the outcome of interest may have multiple attributes including benefits and harms regarding medical, economic, preference-based (quality of life), or time (time spent for care) outcomes. The preference is included by weighing the life by its health-related quality, at any given point in time, and then discounting benefits, harms and cost to reflect the time preference (e.g., preferring a benefit or cost savings now compared to later). In order to compare the alternative choices, the outcome measures are combined in a contrasting result measure. Examples are incremental harm-benefit ratios (IHBR) expressed as number of additional harms to prevent one case of disease or to incremental cost-effectiveness ratios expressed in additional cost per quality-adjusted life year (QALY) gained. However, other combinations are also possible such as the benefit harm trade-off [96].

The perspective adopted may be patient-centered, or focused on the caregiver, the health care provider or payer, in addition to the societal perspective [97]. The perspective of the analysis is crucial for including the corresponding health effects and costs.

#### 3.2.2 The decision-analytic model and assumptions

Decision-analytic models can be used to run computer simulations. Such models are a replicable and objective attempt to mimic the complexity and uncertainty of the real world in a more simple and comprehensible manner. Decision-analytic models should account for events over time and across populations, changing risks, and uncertainty. The purpose of decision-analytic modeling is to estimate the effects of an intervention on valued health consequences and costs. The data implemented in decision-analytic models may be based on evidence from several primary and/or secondary sources and is explained in section 3.2.3 [3], [4], [7], [14], [30], [66], [98], [99].

Several different model types exist that may be combined when appropriate [2], [15], [16], [17], [18], [38], [53], [54], [63], [64], [66], [67], [100], [101], [102], [103], [104], [105], [106], [107], [108], [109]. For relatively simple problems with a fixed time horizon and no time-dependent parameters, decision trees may be suitable. When time is important and influences parameters and events, and where events are repetitive, state-transition cohort (Markov) models may be preferable [63], [64]. However, Markov models follow the Markovian assumption that transition probabilities are independent of prior history. Information about patient history and further characteristics should be included in the definition of simulated health states. If an unmanageable number of health states is required, individual-level state transition microsimulation models (microsimulations) [63], [64] or agent-based models (ABM) may be alternative modeling approaches not limited by the Markovian property [110], [111]. In these models, patient history and other information pertaining to certain simulated individuals can be tracked and updated during the simulation and determine transitions. Agent-based models, discrete event models, or dynamic transition models may also be an option when the model needs to simulate interactions between individuals [112]. In some decision problems, resource constraints or queueing may be a problem, which can be explicitly simulated with discrete event models [2], [38], [66], [67], [100], [101], [102], [103], [104], [105]. It should be noted that sometimes informally, the term ‘cohort model’ is used for modeling groups and the term ‘microsimulations’ or ‘agent-based’ models is used for modeling individual units.

**Figure 3:**
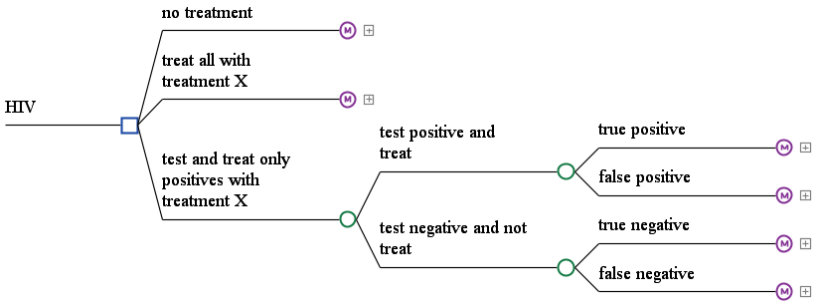
Potential decision tree as start of decision problem in HIV-positive patients

Often, a decision-analytic model is a combination of a short-term decision tree and a long-term disease model. This is explained using an example for such a “hybrid” model combing a decision tree and a Markov state transition model. The decision tree starts with decision options and the first action of the options, which could be a test, a treatment, etc. The recursive disease model is then attached and could be any of the above mentioned models. A typical research question is contrasting treatment strategies to testing and treating depending on test results strategies and no treatment strategies [14], [113], [114], [115], [116], [117]. A potential starting decision tree is shown in Figure 3 (Fig. 3).

**Figure 4:**
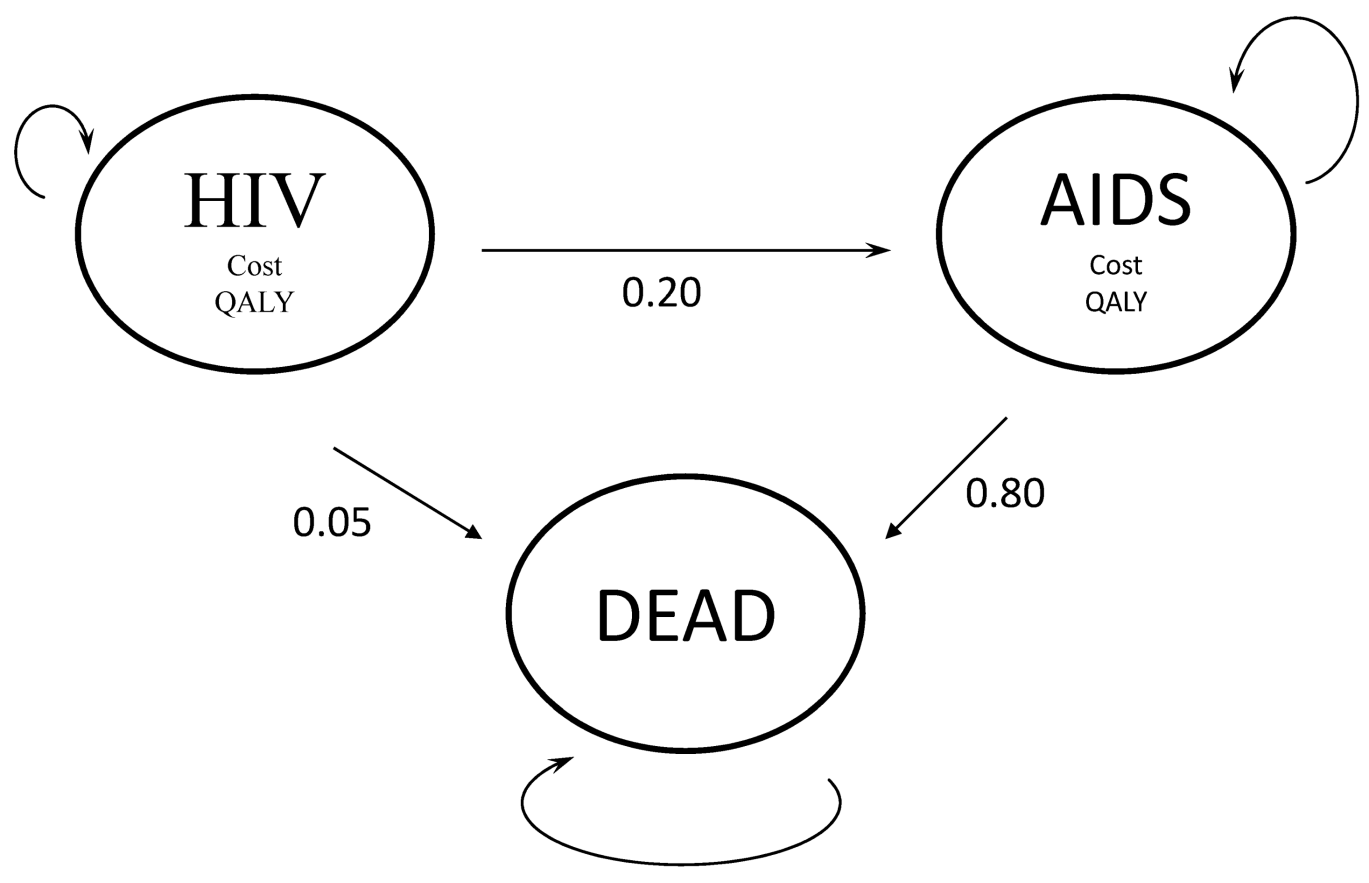
State transition diagram

When structuring a disease model, one has to (1) determine health states, (2) determine transitions, (3) estimate event and transition probabilities, (4) estimate state utilities and costs per time unit, and (5) choose an analytic time horizon. For a Markov model, time is divided into cycles with fixed duration (i.e., cycle length). The basic structure of a model is based on prior knowledge and often visualized and discussed using a state transition diagram as shown in Figure 4 (Fig. 4), which displays the different health states and the possible transitions. During the time dwelling in a given health state, a simulated patient collects benefits and/or harms (e.g., QALYs and costs) and is at risk of moving to another health state based on the characteristics of the current health-state. The Markovian assumption denotes that only the current health state determines the risk of transitioning to another health state. Prior history, that is, prior health states, does not influence that transition probability [110], [111]. A state-transition microsimulation gets around this problem by simulating one individual at a time and gathering (and memorizing) information during the course of disease. This also allows estimating time to pre-specified events. When building discrete event simulation (DES) models, individual history can also be taken into account. However, transitioning is modeled as time to event in contrast to a rate or probability to progress to the next state, as it is done in state transition models [52].

#### 3.2.3 Input parameters

Data used to inform input parameters of decision-analytic models can be derived from prior knowledge, primary (individual-level) data, secondary data such as the published literature and study reports, or – if data are not available – from expert opinion. Primary data would be the first choice, as the analyst has some flexibility to generate the input parameter data in a format that suits the purpose of the model. However, often primary data are not accessible to calculate all transition probabilities over the entire time horizon of the model. The strength of a decision-analytic model is that one can combine evidence from multiple sources and use the findings to make predictions for a different setting (in terms of different time-horizons or similar populations). As secondary data are often presented in a format that cannot be directly used for the model, data need to be transformed. Several methods exist on how to transform or adjust such data in order to serve the model.

In section 6, we briefly describe the main components of model input data and how to transform such data.

#### 3.2.4 Model validation

Models are artificial constructs simplifying the real world and synthesizing evidence with different quality. Therefore, it is important to assess the validity of the model. The ISPOR-SMDM Modeling Good Research Practices Task Force published guidelines on model validation [39], [40]. Five steps of validation are recommended, though in many instances not all five can be implemented. These five steps are face validation, verification or internal validation, cross validation, external validation, and predictive validation. The face validation may be performed by discussing the model structure as well as input parameters and sources with a team of experts. Internal validation is performed by checking the codes and data manipulation process. This can be done by reproducing input data, hand calculation checks and extreme value calculations. Cross validity is provided by comparing the results of a given model with other models analyzing the same problem in the same cohort. External model validation compares the model results with real-world results. Predictive model validation is rarely done. It is comparing model results with prospectively observed events [39], [40].

#### 3.2.5 Performing the analysis

When conducting the analyses of a cohort model, the entire cohort is simulated at the same time, while in microsimulation models, the individuals are run through the model one by one [113], [114], [118], [119]. As mentioned earlier, decision-analytic models may be used to analyze multiple outcomes. Over the time horizon of the analysis, outcomes are accumulated to the total average outcomes. To evaluate the impact of parameter uncertainty on model results and associated conclusions, sensitivity analyses should be performed (see section 3.2.8.).

#### 3.2.6 Model results

Health outcomes and costs of alternative health technologies or treatment strategies are evaluated and compared across strategies. A common combined measure is the incremental cost-effectiveness ratio (ICER). The ICER is calculated by dividing the difference in total costs of alternative technologies by the difference in the chosen measure of health outcome or valued effect (e.g., QALYs). The ICER provides information on extra cost per extra unit of health effect of a new versus standard strategy [120]. Most countries use those ICERs to compare them to ICERs of other treatment options across the health care system. Other countries compare those ICERs only to other treatment options within the same area of indication [121].

#### 3.2.7 Uncertainty analysis

A model is just as good as its input parameters. On the other hand, input parameters themselves are surrounded with uncertainty. Sensitivity analyses are widely requested to test the impact of uncertainty around input parameters and assumptions on the model structure [15], [16], [19], [20], [122]. In the literature, estimates on the mean or median, standard error and 95% confidence intervals for input parameter values are usually provided. In deterministic sensitivity analyses, parameter values are varied within defined ranges or using specified data points. In probabilistic sensitivity analyses, parameter uncertainty (random errors) is described by distributions and considering all relevant parameters at once [25].

However, systematic errors may also be a problem and may also be within the input parameters. As the model is based on secondary evidence, it is dependent on its valid estimation and reporting. When certain results are published more often than others, publication bias may be an issue. Also, decision analysis is interpreting almost all input variables causally. Only unbiased estimates should therefore be included in the model. When the model structure is not correctly specified, systematic bias may occur. The validation process helps to identify those errors.

#### 3.2.8 Limitations and challenges

Breaking down complex decisions to simplified models, is the big asset as well as the main limitation of decision analysis. As Weinstein and Fineberg say [95]:

*Nature is probabilistic*

*And information incomplete*

*Outcomes are valued*

*Resources limited*

*Decisions unavoidable*

A decision-analytic model simplifies the complexity of nature and uses primary evidence to populate the model. This bears potential for bias. First, simplifying nature comes with simplifying assumptions, and is therefore prone to uncertainty and bias. Moreover, some decision-analytic models are relatively complex and need quite extensive data that may not always be available, or data are only available for another population and setting, and therefore, causal interpretation in another context may be questionable. However, despite the complexity and uncertainty in the decision problem, the decision must be made. So, we need aids to structure a decision problem in an explicit and transparent manner. Well-conducted decision-analytic studies clearly lay out the assumptions made and provide extensive sensitivity analyses testing those assumptions.

Furthermore, it should be clear that decision-analytic studies are not providing any new primary empirical evidence. Most decision-analytical studies gather, assess, abstract, and merge published evidence without providing own primary data analyses. As the aim of decision analysis is laying out the entire environment and complexity of the decision problem, empirical data from one source are never sufficient to solve the decision problem. By merging data for several competing outcomes and from several sources, decision-analytic studies provide a structured view on the decision as well as quantitative tradeoff measures such as incremental benefit-harm ratios or incremental cost-effectiveness ratios to explicitly inform about the tradeoff between benefits, harms and costs caused by compared interventions.

Decision-analytic models often value the outcomes by adjusting the life expectancy by the quality of life at each time point and each health state using utility weights. These utility weights express preferences for health states. It is widely debated whether preferences can be applied to entire cohorts, knowing that preferences may differ widely between individuals. Furthermore, it is debated whether preferences can be applied as a constant number assuming that the preference for a given health state is constant. A lot of ongoing research is aiming to improve utility assessment and the application of utilities in decision-analytic models.

Decision analysis follows the utilitarian philosophy of maximizing utilities. However, other aspects may trigger the decision and the optimal choices laid out by the decision-analytic model may not reflect all relevant aspects to be considered by the patient, caregiver, or politician. This does not indicate that the decision maker is irrational but rather that the decision-analytic studies face the difficulty of incorporating all aspects that go into a decision such as ethical, political, or legal concerns. Some aspects may even be conflicting.

It must be noted that decision-analytic modeling according to methodological guidelines and best practice recommendations requires a substantial amount of time and resources [15], [16], [17], [18], [19], [20], [39], [40], [53], [54], [63], [64], [66], [108], [109], [112], [123]. The goal of decision analysis is to explicitly lay out and balance all aspects of a decision and to be transparent with all assumptions and data. To meet this goal, best available evidence must be detected, assessed, abstracted, and combined. In addition, the model must be developed, calibrated, validated, and analyzed. This is a time-consuming undertaking and may take several months or years.

#### 3.2.9 Software

Decision-analytic software for model development ranges from visual interactive modeling software with graphical user interfaces to high-level programming languages. Programming languages are most flexible with respect to code writing and run-time optimized code but require in depth programming skills. Visual interactive modeling software supports model implementation and model visualization for decision makers. Software such as TreeAge, AnyLogic, Arena, Simul8, Vensim or others are specialized in different modeling approaches. In parts, this software supports transformation of input parameter values (e.g., rates to probabilities or fitting of distributions to underlying data like in survival analysis). However, input parameter values or risk functions that determine transitions and the pathway of patients are mainly determined upfront using statistical software packages. The statistical programming language R is increasingly applied for data analysis and to build decision-analytic models, since decision-analytic packages are being developed to support model implementation and analyses [124], [125]. General programming languages such as Java, C++ or Python are applied especially for complex individual level simulations (state transition microsimulation, DES, agent-based models).

## 4 Discussion

### 4.1 Summary

This scoping document summarizes the methods of causal inference in epidemiology and health decision science. Both areas aim at comparing different intervention strategies following a counterfactual approach and estimating a valid causal effect on one or more outcomes of interest. Both methods aim at generating evidence to guide decision makers in complex decision making processes. In this paper, we described each method separately using a common language and selected case examples. This should aid and support understanding and appraising studies that apply these methods.

Causal inference is the methodology using empirical data to draw conclusions on one intervention on one or more disease outcomes of interest over a specified, often limited, time horizon. Health decision science methods are usually applied later in the decision process, looking at health policy questions where aspects besides clinical elements are being considered and reflected in the outcomes. Patient preferences are part of the analysis that is based on multiple sources and very often secondary data. The time horizon is often longer than the one of clinical studies or observational databases and simulation is used to extrapolate outcomes or link evidence from short-term RCTs and long-term observational data. Models in health decision science studies depend on unbiased model input parameters. These input parameters include effect estimates that must be drawn from epidemiologic causal inference studies.

There are several features that are critical for both causal inference and decision-analytic modeling. In both fields, the population of interest must be chosen or defined. Then the causal structure of the decision problem must be discussed and implemented. Data must be collected: in the case of causal inference, the epidemiologists may start a cohort study; in the case of decision-analytic modeling, the decision analysts will start performing systematic literature reviews to gather evidence on the model parameters. Finally, both methods estimate an effect of the intervention.

### 4.2 Context to literature

Some published literature exists comparing the performance of both methods using specific examples [126], [127]. The focus of these studies regarding the decision-analytic methods is the performance of ABM. The authors question how decision-analytic models can transform retrospectively gained knowledge into the future. This is an important aspect of decision science in general and decision making itself. How can prior knowledge help to make the best possible decision? The literature looks at very detailed parts of decision analysis and is meant for “statistically minded researchers”. Further literature describes the difficulties of ABM when being applied in areas outside of the areas from which its parameters were obtained from [128]. These aspects raised in the literature are very important, valuable and complex. The intention of our scoping document is different. We believe that explaining both methodological areas to a wider audience is an important if not necessary first step providing the basis for discussion. This understanding can then be used to make decisions when and how causal inference and decision-analytic modeling can be used and combined in the decision making process.

Often decision-analytic models base their estimated transition probabilities on epidemiological studies that report associations without claiming a causal relation. This is discussed with three examples: (1) using prediction scores, (2) transferring data to other populations and time horizon, (3) risk of biases in effect measures from RCTs, (4) using regression models as model input parameters.

**Example 1:** The Framingham Heart Study is a well-known study predicting the 10-year risk for cardiovascular events [129]. The prediction formula is often used in decision-analytic models to calculate the risk of coronary heart disease (CHD) based on subjects’ clinical and other characteristics. For example, intervening on lifestyle is of interest and decision-analytic models are constructed using the risk score from the Framingham heart study [130]. However, regression methods as used in the Framingham Heart Study work well for the prediction of CHD risk, but we need to be careful when interpreting these associations causally. On the causal pathway of body mass index (BMI) to coronary heart disease (CHD) for example, physical activity may be a time-dependent confounder (i.e., a confounder that simultaneously acts as an intermediate step). In these situations, g-methods must be applied to validly estimate the causal effect of change in BMI on the occurrence of CHD [11].

Of note, it has taken nearly two decades from the development of the theoretical concept of the g-formula by Robins [61] until this causal inference method was first applied by his doctoral student Siebert to real world data in his dissertation under the supervision of Robins and co-supervision of Hernán in a collaboration project with WHO aiming to assess the causal effect of interventions on multiple risk factors of CHD [11], [78].

**Example 2:** Murray and colleagues compared the performance of decision-analytic microsimulation (here called “agent-based modeling”) and the application of the g-formula in estimating the 12-month mortality in HIV-positive patients [128]. They concluded that both modeling techniques performed well when the input parameters of the agent-based model are estimated within the same cohort the model is reflecting. However, when estimates are being extrapolated to other populations or time horizons with different underlying risk factors, the agent-based modeling may result in bias.

**Example 3:** Not only observational studies are at risk of biases. Due to ethical and practical reasons, some RCTs allow switching to the active treatment when disease progression is observed. However, when randomization is violated, risk of time-dependent confounding is an issue [131]. The National Institute for Health and Care Excellence (NICE) in the UK has published several appraisals that come to very different cost-effectiveness ratios when using input parameters estimated using g-methods or traditional (associational) methods [57], [132], [133], [134], [135], [136]. Those different input parameters would have led to very different decisions.

**Example 4:** A well-performed and transparently described decision-analytic diabetes microsimulation model [137], [138] estimates the transition probabilities for risk factors and disease complications based on (traditional) regression coefficients. The regression analysis is conducted using a large observational cohort. This author group was able to use this cohort study to estimate each transition parameter. However, when developing the regression model for the use of estimating transition probabilities for a decision-analytic model, one has to carefully consider whether the estimated parameters can be interpreted causally.

### 4.3 Limitations

This scoping document has several limitations. The selection of textbooks and articles included in this scoping work was primarily based on the long-term experience of the expert authors and no systematic literature search was performed. However, an unsystematic search was used to address issues related to the combination of causal inference and decision-analytic modeling.

In this scoping document, we did not cover all aspects of the methodological areas of causal inference and health decision science. Parts that were not explicitly discussed are complications with compliance, selection bias, unmeasured confounding or immortal time bias. These issues are debated in the field of causal inference as well as among decision analysts [43], [47], [50], [139], [140], [141]. Also, details and problems of causal model specification were not discussed in detail. In this scoping review, we did not cover decision-analytic models with interactions between individuals. This is especially relevant when assessing measures against the spread of acute infectious diseases. The terms “public health” and “modeling” likely became known over the entire globe during the COVID-19 pandemic. In addition, decision-analytic techniques such as discrete-event simulation (DES) exist that are especially useful for research questions looking at scarce resources and issues of queueing (waiting lines) [33], [52], [53], [54], [65], [142]. We did not discuss these methods in this scoping review. The full scope of causal inference and health decision science is enormous and growing daily, and the scope of this review was to provide a basic overview of these methodological approaches. Hence, our focus was on a description of the concepts and an overview of commonly applied models. For more detailed and complex information, the corresponding text books and methodologic papers should be consulted [10], [15], [16], [17], [18], [19], [20], [23], [32], [39], [40], [46], [47], [49], [50], [53], [54], [55], [62], [63], [64], [66], [73], [108], [109], [143], [144].

Another limitation of this scoping document is that the examples from the literature have not been based on a systematic review but have been chosen based on their ability to provide a formal and intuitive understanding of the causal question and the relevance to causal inference as well as decision-analytic concepts. The examples came from different diseases and covered different important aspects. The examples showed that in a decision-analytic model, parameters for risk factors that are influenced by the intervention of interest should be kept in the causal equations for outcomes mediating the effect of these interventions. This may be difficult when the estimates come from regression models with selection criteria following mere statistical (e.g., p-value based) rules [12], [35]. Further, the examples showed that even data from RCTs need to be carefully interpreted when randomization in the trial was violated, for example by treatment switching, in particular if this switching would not be possible in one of the counterfactual worlds. Another example pointed to a study that sensitized for potential problems when transferring data from one population to another and from one time horizon to another. We wanted to show that transition probabilities at different positions in decision-analytic models must be seen as causal model input parameters. Hence, the modeler must carefully watch and question such links.

An interesting field is the validation of causal inference analyses and decision analyses with external and independent data. Increasingly, causal analyses of observational data are compared to clinical trials. The data from the causal analysis are used to emulate a clinical trial based on the target trial approach [50], [55], [144]. If the results from a trial are available, the two study types can be compared. Decision analyses usually have a longer time horizon, and therefore, the real future is often suggested as a gold standard to assess the validity of the models. The latter approach is mostly not feasible, as health care changes, the behavior of people changes, and other circumstances may change, which will all lead to different results.

Ewald and colleagues [41] performed a systematic review and meta-analysis comparing the results from 141 RCTs (120,669 patients) with those of 19 MSM-studies (1,039,570 patients) and concluded that the results of the MSM studies differed from those of the RCTs, and “caution is required when nonrandomized “real world” evidence is used for healthcare decisions” [41]. However, as standardization for the different study populations in RCTs and observational studies is not possible based on mere secondary data, it is not known how much of the difference between RCTs and observational studies is explained by different underlying study populations or by different study designs and (residual) confounding in the observational studies.

### 4.4 Outlook and future trends

Causal inference has come a long way since Robin’s milestone article in 1987 [61] and meanwhile has made its way into mainstream science and epidemiologic textbooks [145]. However, most applications have been in the areas of medicine and epidemiology, with IPTW being the most dominant estimation technique [34]. As IPTW is known to be potentially sensitive to positivity violations and model misspecification, an uptake of modern doubly robust estimation techniques (e.g., TMLE), in conjunction with machine learning, can be expected. Avoiding human error in modeling is certainly a dominant trend in the current research field. For these estimators to perform well, good choices for appropriate machine learning algorithms have to be made [24], [146]. One promising approach is the highly adaptive LASSO estimation [147]. To avoid the problem of positivity violations, the use of stochastic interventions has been proposed [148]. While a focus on computational trends is meaningful, the choice of appropriate correction methods in randomized trials [49] and the development of standards for explaining and justifying DAGs are certainly other areas of high research priority.

Another field in which we expect more applications of g-methods is in RCTs with treatment switching. Several methodological approaches have been developed in the last years [136], [149], [150], [151], and HTA agencies and networks have included the use of g-methods for adjusting for treatment switching in its HTA recommendations [57], [131]. We will hopefully see more comparisons of (1) causal inference studies and RCTs, (2) causal inference studies with studies using the traditional regression methods, (3) causal inference studies and decision analyses. And we will hopefully also see more collaboration of these two fields. Due to the integration of causal inference courses in scientific societies such as the Society for Medical Decision Making (SMDM), the International Society for Pharmacoeconomics and Outcomes Research (ISPOR) etc., there will be more cases in which decision modelers critically judge their model parameters from the literature but also use DAGs themselves when generating a causal decision-analytic model.

It is expected that we will see more educational efforts on causal inference, decision-analytic modeling, but also in the combination of both fields.

Perhaps most importantly, the causal target trial approach may slowly enter the field of medicine and public health, and guide researchers to design their observational studies well.

Finally, the new partnership of causal inference and health decision science will be extended by a third party: machine learning in causal inference and modeling allowing for applying these methods in big data.

## 5 Conclusions

Our scoping document shows that both causal inference and health decision science are important components of a comprehensive and valid health policy decision making process. Both methodological areas are aiming for providing evidence to optimally guide evidence-based decision making. However, the approaches, strengths and limitations of these methods differ. Causal inference uses empirical individual level data to draw causal conclusions on the effect of an action on (usually) a single or selected outcome. Decision science, on the other hand, aims at integrating all aspects of a decision and at comparing the effect of two or more strategies on several integrated outcomes. It combines outcome measures, for example, life expectancy and quality of live, and synthesizes data from several sources. Both disciplines use complex computer models that need to be correctly specified and sometimes lack acceptance. Both methods have potential for bias. The typical biases in causal inference analyses are those that are common in observational database analyses including confounding, immortal time bias, selection bias and others. In decision-analytic studies, the risk of bias is mostly due to false assumptions, oversimplified model structures, biased input parameters, or insufficient consideration of uncertainty. Basic knowledge on both of these methods is necessary to decide when and how these methods are applied. Importantly, both methods should be combined when developing health decision guidance and recommendations, for example in clinical guidelines, health technology assessment reports, reimbursement decision dossiers, patient information and shared decision making processes. Further research should contrast these methods and identify interfaces for synergies, both in research and education.

## 6 Technical notes on input parameters

The following description is based on a former project funded by the Austrian Research Promotion Agency (FFG).

### 6.1 Transition probabilities for disease progression and mortality

In order to estimate the long-term effects of treatments or treatment strategies, the modeler simulates the underlying course and progression of disease. Data on the natural course of the disease and its progression may be derived from several sources, epidemiologic cohort studies, registries, claims data, and other retrospective databases. Under certain circumstances, short-term progression data may also be derived from clinical trials.

Relative straight forward measures are disease frequency measures, such as prevalence (i.e., the proportion of individuals in a population who have the condition at a particular time) and incidence (i.e., the risk of contracting the disease or developing some new condition within a particular time). However, the correct use of risk measures in the model is essential for valid model predictions. One has to carefully differentiate and appropriately transform the data if necessary to implement valid probability estimates for the transitions [152], [153].

In many instances, studies provide survival data and Kaplan Meier curves. Those data can also be included into a decision-analytic model. Methods of survival analysis can be applied to convert such data into rates to include them into a state transition model, or fitted survival curves can be used to populate a DES model [7], [17], [18], [53], [54], [63], [64], [154], [155].

The risk of clinical events including mortality might be increased by the presence of risk factors. Risk factors can be taken into account by stratifying the cohort according to the risk factors and estimating the event rates or mortality rates as described above. However, many clinical studies describe the influence of risk factors as relative risk, odds ratio or hazard rate. All of these estimates can be superimposed on the baseline risk. However, the impact of risk factors has been evaluated in a specific study population that may differ from the modeled hypothetical cohort. Techniques exist to standardize the estimators to the cohort of interest.

### 6.2 Effects of intervention 

The recommendation of the ISPOR Task Force 2003 [66] on how to incorporate treatment effects into the model is to derive estimates of relative risks or odds ratios and superimpose these on baseline probabilities. This can be done as it is described for the risk factors. However, the literature does not always provide these estimates from a head to head comparison. In these instances, indirect treatment comparison meta-analyses of all studies may be an option, where the effects are being pooled over several studies. Studies that provide model input parameter values should be selected carefully.

If an intervention works through influencing a risk factor (e.g., statins reduce cholesterol) then it is crucial that the risk factor effect (e.g., relatives risks or odds ratios) can be interpreted causally. Therefore, this risk factor effect must be estimated with the appropriate causal inference methods (e.g., controlling for confounding).

**Figure 5:**
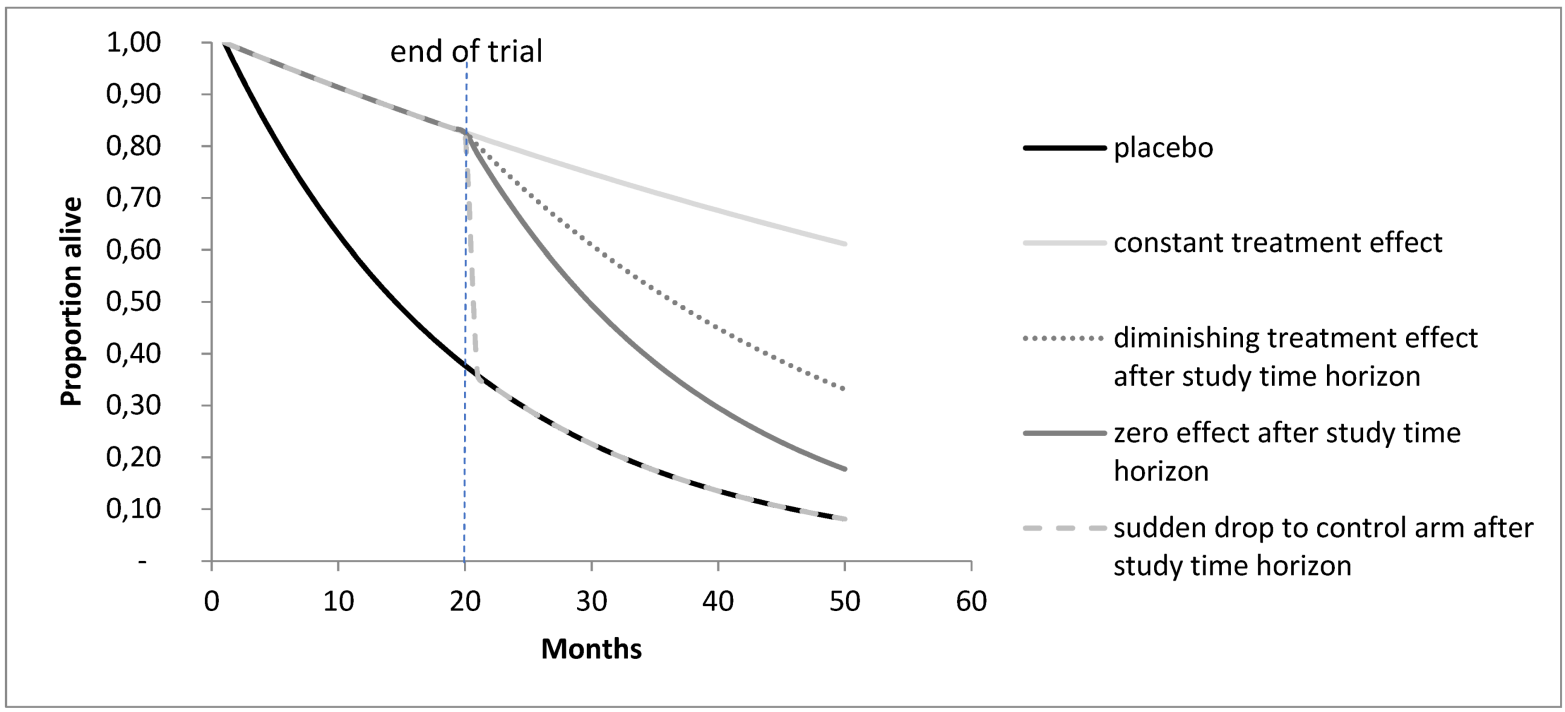
Extrapolation of treatment effects

Another issue to consider when estimating the treatment effect is the extrapolation of the effect beyond the time horizon of the trials. Basically, four different assumptions for the extrapolation of the treatment effects can be made: (1) constant treatment effect, (2) diminishing effect over time (“fade out”), (3) zero effect after end of study, or (4) sudden drop to control arm (“stop and drop”). They are shown in Figure 5 (Fig. 5). The choice of assumption should be guided by the disease and the treatment.

### 6.3 Performance of diagnostic tests

**Figure 6:**
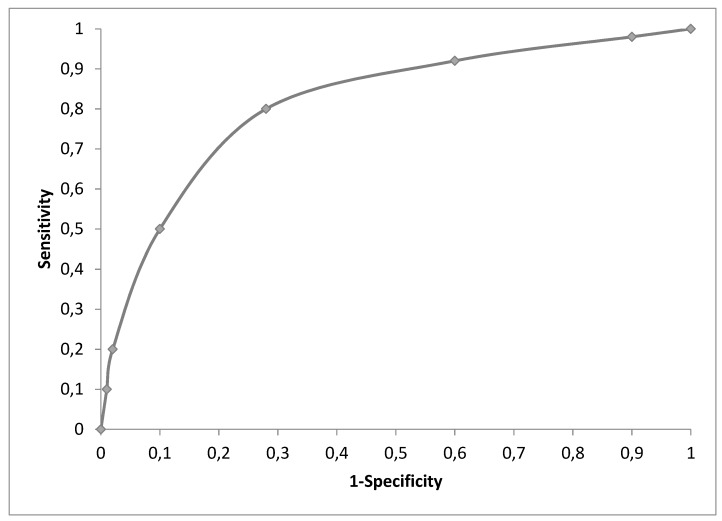
Receiver operating characteristic (ROC) curve

For decision-analytic models that include diagnostic tests, it is important to include the test performance characteristics properly into the model [96]. As most tests are imperfect, and both false positive and false negative tests may have their clinical consequences, it is important to include the test accuracy into the model. The pretest probability of disease is the probability of having the disease given the information prior to performing the test. This might or might not be the prevalence. The posttest probability of disease is the probability of having the disease, given all pretest and test information; it can be calculated in several ways. The sensitivity is a test characteristic that is often reported and is defined as the probability of a positive test, given that the disease is present. Specificity is defined as the probability of a negative test, given that the disease is not present. In contrast to dichotomous tests, multilevel or continuous tests have more than two possible outcomes. In theory, the test could be made dichotomous at each level, and for each outcome the described test characteristics could be calculated. To visualize the trade-off between sensitivity and specificity, a graph called receiver operating characteristic (ROC) curve is shown. The graph shows the relationship between test characteristics; that is, sensitivity is plotted against 1-specificity for each possible test-result cut-off. At the extreme values, either sensitivity or specificity are very high or very low (Figure 6 (Fig. 6)).

### 6.4 Utilities

In the literature, several methods exist for measuring the health-related quality of life (HRQoL). In decision-analytic models, HRQoL is usually incorporated as utilities. A utility is a global measure of the preference concerning a health state, reflecting all aspects of the health state, measured on a ratio scale and using the length of life as the metric for measuring the preference [5], [156]. As HRQoL is also depending on socioeconomic and cultural aspects in a specific country, utility data retrieved from the literature and transferred to another context should be treated with caution. Therefore, most guidelines and recommendations for good practice in cost-effectiveness modeling recommend that utilities should be generated directly from primary data using standard methods such as standard gamble, time trade-off or preference-based generic instruments [157].

### 6.5 Costs

Depending on the perspective and country of the analysis, different types of costs must be included in the decision model. Different HTA agencies published different guidance on costing approaches to be used for cost-effectiveness assessments [21], [22], [158].

In general, the costs should be assessed following the 3-step micro-costing approach, that is, identification, measurement, and valuation of resource use. However, some instances might justify a gross-costing approach. Only the value of those goods and services that change because of the intervention should be considered. And the prices used in the analysis should reflect the prevailing prices in the location where the intervention is or will be implemented. Opportunity costs are often well reflected in prices. Where this is not the case, adjustments should be made. Wages are generally an acceptable measure of time cost, while age- and gender-specific wages should be used to best reflect the target population. Unpaid services provided by volunteers or family members should be estimated using hourly wages of a corresponding individual that is working for pay.

All costs included into the analysis should be updated to constant cost units, using the consumer price index and, where appropriate, the medical component of it [97].

## Notes

### Acknowledgments

We thank Nikolai Mühlberger for his support and careful thoughts on parametrization of decision-analytic models.

### Competing interests

Felicitas Kühne, Michael Schomaker, Igor Stojkov, and Uwe Siebert teach Causal Inference courses in the HTADS Program on Continuing Education at UMIT TIROL (http://www.htads.org).
